# Resolvin D2 Reduces UVB Skin Pathology by Targeting Cytokines, Oxidative Stress, and NF-κB Activation

**DOI:** 10.3390/antiox14070830

**Published:** 2025-07-06

**Authors:** Ingrid C. Pinto, Priscila Saito, Camilla C. A. Rodrigues, Renata M. Martinez, Cristina P. B. Melo, Maiara Piva, Clovis M. Kumagai, David L. Vale, Telma Saraiva-Santos, Allan J. C. Bussmann, Marcela M. Baracat, Sandra R. Georgetti, Fabiana T. M. C. Vicentini, Waldiceu A. Verri, Rubia Casagrande

**Affiliations:** 1 Department of Pharmaceutical Sciences, Centre of Health Science, Londrina State University, Avenida Robert Koch, 60, Londrina 86038-350, PR, Brazil; 2 Department of Immunology, Parasitology and General Pathology, Centre of Biological Sciences, Londrina State University, Rodovia Celso Garcia Cid, Km 380, PR445, Cx. Postal 10.011, Londrina 86057-970, PR, Brazil; 3 School of Pharmaceutical Sciences of Ribeirão Preto, University of São Paulo, Av. Café, Ribeirão Preto 14048-900, SP, Brazil

**Keywords:** pro-resolution lipid, antioxidant, UV radiation, inflammation

## Abstract

UVB skin pathology is initiated by reactive oxygen species (ROS), differentiating this condition from other inflammatory diseases involving first the immune cell activation by danger or pathogen molecular patterns followed by oxidative stress. Resolvin D2 (RvD2) has been found to reduce inflammation in preclinical models. However, whether or not RvD2 reduces skin pathology caused by UVB irradiation is not yet known. Therefore, the efficacy of RvD2 on skin pathology triggered by UVB irradiation in female hairless mice was assessed. RvD2 (0.3, 1 or 3 ng/mouse, i.p.) was found to protect the skin against UVB inflammation, as observed in the reduction in edema (46%), myeloperoxidase activity (77%), metalloproteinase-9 activity (39%), recruitment of neutrophils/macrophages (lysozyme^+^ cells, 76%) and mast cells (106%), epidermal thickening (93%), sunburn cell formation (68%), collagen fiber breakdown (55%), and production of cytokines such as TNF-α (100%). Considering the relevance of oxidative stress to UVB irradiation skin pathologies, an important observation was that the skin antioxidant capacity was recovered by RvD2 according to the results that show the ferric reducing antioxidant power (68%), cationic radical scavenges (93%), catalase activity (74%), and the levels of reduced glutathione (48%). Oxidative damage was also attenuated, as observed in the reduction in superoxide anion production (69%) and lipid hydroperoxides (71%). The RvD2 mechanism involved the inhibition of NF-κB activation, as observed in the diminished degradation of IκBα (48%) coupled with a reduction in its downstream targets that are involved in inflammation and oxidative stress, such as COX-2 (66%) and gp91^phox^ (77%) mRNA expression. In conclusion, RvD2 mitigates the inflammatory and oxidative pathologic skin aggression that is triggered by UVB.

## 1. Introduction

Skin cancer is an important disease and the process of photo-carcinogenesis triggered by solar ultraviolet (UV) radiation repetitive exposure is considered a primary cause of it [1,2]. Ultraviolet irradiation comprises three bands with different wavelengths: UVC (280–100 nm), UVB (320–280 nm), and UVA (400–320 nm) [2,3,4]. UVB irradiation is also the main cause of sunlight’s harmful effects. The pathological events in the skin involve direct damage through DNA mutations and oxidative stress. Specifically, the superoxide anion is an initiating reactive oxygen species (ROS) whose production occurs upon UV-triggered mitochondrial electron leakage from the respiratory chain complexes I and III [5]. The evidence supports the idea that superoxide anions cause edema formation, leukocyte recruitment, cytokine production, and further boosts oxidative stress [6,7,8,9,10], leading to inflammatory and oxidative skin pathologies [11,12,13,14].

The excessive ROS production observed upon UVB irradiation surpasses the endogenous antioxidant defenses capacity, leading to an imbalance known as oxidative stress that threatens the integrity and function of cellular structures. In addition, oxidative stress leads to the activation of various signaling pathways such as nuclear factor-κB (NF-κB), thus promoting the transcription of genes involved with the inflammatory response, such as cyclooxygenase-2 (COX-2, an enzyme that produces protaglandins), gp91^phox^ (a subunit of NADPH (nicotinamide adenine dinucleotide phosphate) oxidase, an enzyme that produces superoxide anion), and cytokines (molecules that orchestrate immune cell function) [15,16,17,18,19]. These molecules have a role, for instance, in edema formation and the recruitment of inflammatory cells to a lesion site. The inflammatory process onset leads to additional free radical production, acting as a defense mechanism; accordingly, this process becomes a vicious cycle that generates tissue pathologies [20,21].

Resolvin D2 (RvD2) is classified as a specialized pro-resolution lipid mediator (SPM) that can be identified in inflammation sites at the resolution phase. Docosahexaenoic acid (DHA) is a dietary omega-3 polyunsaturated fatty acid that serves as a substrate to RvD2 synthesis [22,23]. Pro-resolution mediators have three main effects: they are anti-inflammatory, pro-resolution, and non-immunosuppressive [24]. RvD2 treatment reduces tissue injury in varied conditions ranging from spinal cord injury [25], age-related hepatic injury [26], allergic eosinophilic lung inflammation [27], mucin-layer-dependent ocular homeostasis [28], ischemic stroke [29], brain injury caused by subarachnoid hemorrhage [30], cellular death caused by middle cerebral artery occlusion and reperfusion [31], thrombosis and organ necrosis secondary to burn wounds [32,33,34,35], and neuronal injury [36].

Interestingly, RvD2 treatment inhibited oxidative stress in the retina of diabetic mice in the streptozotocin model [37]. RvD2 increased the levels of superoxide dismutase and reduced glutathione (GSH) as well as decreased lipid peroxidation and DNA damage in diabetic mouse retina [37]. UVB causes oxidative stress, which is part of its pathological mechanisms [2]. However, the activity of RvD2 against UVB irradiation skin pathology has not been assessed as far as we are aware of. Therefore, the present study investigated the activity and mechanisms of RvD2 against UVB irradiation skin pathology.

## 2. Materials and Methods

### 2.1. Chemicals

RvD2 with purity ≥ 95% was obtained from Cayman Chemical (Ann Arbor, MI, USA). Brilliant blue R, reduced glutathione (GSH), 2,2′-azinobis (3-ethylbenzthiazoline-6-sulfonic acid) (ABTS), 2,4,6-tripyridyl-S-triazine (TPTZ), Trolox, nitroblue tetrazolium (NBT), 5,5′-dithiobis (2-nitrobenzoic acid) (DTNB), o-dianisidine, and bisacrylamide were obtained from Sigma-Aldrich (St Louis, MO, USA). Tert-butyl hydroperoxide was obtained from Acros (Pittsburgh, PA, USA). Hydroxymethyl aminomethane (Tris) was obtained from Amresco (Solon, OH, USA). Acrylamide and sodium dodecyl sulfate (SDS) were obtained from Invitrogen (Vilnius, Lithuania). Immunoenzymatic assay kits (ELISA) for cytokine dosing were obtained from eBioscience (San Diego, CA, USA).

### 2.2. Experimental Groups

The experiments were performed using female adult hairless mice (HRS/J) weighing 30 g. The mice were kept in a room with a controlled temperature (22 ± 2 °C) located in the University Hospital of Londrina State University. The animals were housed under 12/12 h light/dark cycles with water and food at will. Hairless mice have skin that is more similar to human skin in comparison with furry mouse strains. Like other mouse strains, the male hairless mouse is more aggressive than the female hairless mouse and fights to establish hierarchy cause skin physical damage and inflammation. As a result, using female hairless mice avoids unexpected reduction in the number of subjects that could be caused by fight-derived skin damage. The project was approved by the Animal Ethics Committee (CEUA) of the State University of Londrina, following its rules (CEUA process number 1447.2015.10 approved on 19 March 2015).

The animals were randomly (through block randomization) assigned to five experimental groups containing 6 animals each, based on prior studies [13,14]:

(1) Group that was non-irradiated and saline-treated.
(2) Group that was irradiated and saline-treated control group.
(3) Group that was irradiated and treated group at a 0.3 ng/mouse dose of RvD2.
(4) Group that was irradiated and treated group at a 1.0 ng/mouse dose of RvD2.
(5) Group that was irradiated and treated group a 3.0 ng/mouse dose of RvD2.

Mice were treated with a final volume of 200 µL containing titrated concentrations of RvD2 (0.3, 1.0, and 3.0 ng/mouse) or only the vehicle (sterile saline) used to dilute the lipid mediator intraperitoneally (i.p.) 1 h before beginning of radiation. The dose of RvD2 was selected based on the therapeutic effects previously described [13,14,38,39] and in the dose–response experiments performed in this study. The experimenters were blinded.

### 2.3. Model of Induction of Skin Lesion by UVB Irradiation

A UVB fluorescent lamp (PHILIPS TL/12 of 40 W RS from MEDICAL-NETHERLANDS) was used to induce acute inflammatory process and oxidative stress. The lamp range of radiation was 270 to 400 nm reaching 313 nm as a peak of maximum emission and was coupled inside a rectangular wooden box developed specifically for the induction of cutaneous lesions and was placed 20 cm above the animals. The irradiance of this lamp was measured using a radiometer (IL 1700) with detectors for UV radiation (SED 005) and especially for UVB (SED 240) [40], making it possible to standardize that a five hour, thirty minute exposure reached the dose of radiation of 4.14 J/cm^2^ [12,40].

The euthanasia was performed using a 5% isoflurane anesthesia; afterwards, the dorsal skin samples were collected. The first point of sample collection was 12 h after exposure to UVB irradiation. This time point was selected in prior studies as the ideal for assessing skin edema, MPO activity, MMP-9 activity, LysM-GFP^+^ cell fluorescence detection, histology, and antioxidant parameters (e.g., FRAP, ABTS and GSH levels). In another set of experiments, after terminal anesthesia, samples were collected 2 h after exposure to UVB. This second set of samples has been standardized for the tests of catalase and superoxide anion production (NBT assay). In a third set of experiments, after terminal anesthesia, samples were collected 4 h after the end of the irradiation. This last set of samples has been standardized to assess cytokines production, lipid peroxidation, mRNA expression by RT-qPCR and IκBα by Western blot. In these last-mentioned time points (2 and 4 h), the animals were anesthetized with 5% isoflurane followed by decapitation [40,41]. Skin samples were stored separately for each different test at −80 °C, except for the skin edema test that was performed shortly after collection; for the histopathological evaluation, the samples were stored in 10% formaldehyde solution after collection until processing. The time points for sample collection were determined in standardization assays and according to previous studies [40,41].

### 2.4. Skin Edema

The UVB excessive exposure of the skin induces edema formation by increasing vascular permeability and the consequent leakage of interstitial fluid into the epidermis [42]. For this test, mice dorsal skin samples were collected using a fixed area mold (5 mm diameter). Skin weight (mg of skin) was compared among the negative control group, the positive control group, and the treatment groups [12].

### 2.5. Myeloperoxidase Activity (MPO)

The activity of the enzyme MPO represents an indirect test of leukocyte recruitment to the lesion site after UVB irradiation [12,40]. Skin samples were added to a solution with a pH 6.0 constituted of 0.5% hexadecyltrimethyl ammonium bromide in 50 mM phosphate buffer, and then processed with the aid of the Tissue-Tearor homogenizer Biospec 985370 (Bartlesville, OK, USA) and subsequently centrifuged (16,100× *g* for 2 min at 4 °C). For the colorimetric reaction, 30 μL of the supernatant from each sample was added together with 200 μL of a deionized water solution containing 0.0167% ortho-dianisidine, 0.05% H_2_O_2_, and 10% 0.05 M phosphate buffer. MPO activity of the samples was determined spectrophotometrically at 450 nm (EnSpire, Perkin Elmer, Waltham, MA, USA) and compared to a standard curve of neutrophils with known concentration. The results obtained were expressed in the number of neutrophils/mg of skin [40].

### 2.6. Determination of the Activity/Secretion of Metalloproteinase-9 (MMP-9)

MMP-9 is a proteolytic enzyme that degrades important components of the extracellular matrix such as collagen, and its expression is increased through exposure to UVB irradiation [43,44]. The activity of this protease can be determined using an assay called zymography, which detects the degradation of the substrate (gelatin) by MMP-9 in a sodium dodecyl sulfate–polyacrylamide gel [45]. Samples were homogenized with Tissue Tearor-Biospec 985370 (Bartlesville, OK, USA) in a ratio of 1:4 in Tris/HCl phosphate buffer set at a concentration of 50 mM and pH 7.4 with calcium chloride (CaCl_2_). This buffer also contained phenanthroline, phenylmethylsulfonyl fluoride, and N-ethylmaleimide (1%), which are protease inhibitors. The homogenates were doubly centrifuged at 12,000× *g* for 10 min at 4 °C. Aliquots of 30 μL of the supernatant from the samples were diluted in 6 μL of Tris/HCl buffer (pH 6.8) containing glycerol (20%), sodium dodecyl sulfate (4%), and 0.001% bromophenol blue. After this preparation step, samples were applied in the gel (13.5% acrylamide and 0.025% gelatin). After completion of the run, the polyacrylamide gel was washed with 2% Triton X-100 solution for 1 h. The next step was an incubation for a total period of 16 h at 37 °C, which used a Tris/HCl buffer in a concentration of 50 mM and pH 7.4 containing 10 mM CaCl_2_ and 0.02% sodium azide. The last step before the analyses was the staining of the gel, which applied a brilliant blue solution at a concentration of 0.05% and washed with a 20% acetic acid solution for the visualization of the bands. The proteolytic activity was analyzed using the ImageJ^®^ program (NIH, Bethesda, MD, USA) to compare the intensities of the bands between the groups.

### 2.7. Fluorescence Detection of LysM-GFP^+^ Cells

LysM-eGFP^+^ mice were shaved 48 h before irradiation. The collected dorsal skin tissue samples were embedded in 4% PFA for 24 h, transferred for an additional 24 h to a sucrose solution at 30% concentration, and to 1:1 sucrose solution at 30% concentration: O.C.T. and final inclusion in O.C.T. The tissue was sectioned (10 μm) and the sections were handled for the observation of fluorescence under the confocal microscope (Leica TCS SP8, Leica, Wetzlar, Germany) with a 5× objective. One field per sample with an indication of neutrophil recruitment to the skin tissue was chosen and reported as the percentage of eGFP fluorescence intensity [13]. Images were examined using Leica EL6000 software (Leica, Wetzlar, Germany).

### 2.8. Histopathological Evaluation by Optical Microscopy

For the histopathological analysis of the skin, the samples were collected and fixed in 10% formaldehyde until they were processed. Subsequently, they were dehydrated in baths of ethanol solutions with increasing concentrations (70%, 95%, and 100%), diaphanized with xylol, and embedded in paraffin wax. Hematoxylin–eosin (H&E)-stained sample sections measuring 5 μm were analyzed using light microscopy at 40 and 100x magnification for the determination of epidermal thickness [46] and the number of apoptotic keratinocytes [47], respectively. In addition, tissue sections were also stained with toluidine blue for the determination of mast cells with 40× magnification. Analyses were performed using Infinity Analyze (Lumenera^®^ Software, Carlsbad, CA, USA). Finally, sections of the tissues were stained with Masson’s trichrome. This staining aimed to analyze possible changes in collagen fibers (10× magnification) and were analyzed using Image J software version 1.52q (NIH, Bethesda, MD, USA) [48].

### 2.9. Dosage of Proinflammatory Cytokines TNF-α, IL-33, and IL-1β and Anti-Inflammatory Cytokines TGF-β and IL-10

Cytokine quantitation was performed using sandwich-type enzyme-linked immunosorbent assay (ELISA) technique and performed according to the manufacturer’s instructions (eBioscience, San Diego, CA, USA) [49]. The reaction was determined spectrophotometrically at 450 nm (Multiskan GO, Thermo Scientific, Waltham, MA, USA) and the results were compared using a standard calibration curve provided by the kit; results were expressed as pg of cytokines/mg skin.

### 2.10. Evaluation of Ferric Reducing Antioxidant Power (FRAP)

A FRAP assay measures the ability of a skin sample in reducing ferric 2,4,6-tripyridyl-S-triazine (TPTZ) and this reaction leads to color changes through the action of antioxidants that donate electrons to the reaction [50]. Skin samples were homogenized in 400 μL of KCl (1.15%) with the aid of Tissue-Tearor Biospec 985370 (Bartlesville, OK, USA) and centrifuged at 1000× *g* for 10 min at 4 °C. For the reaction, 30 μL of the homogenate supernatant and 150 μL FRAP reagent (10 mM TPTZ in 40 mM HCl with 20 mM hexahydrate ferric chloride and 0.3 mM acetate buffer pH 3.6) were used. This solution was incubated at 37 °C for 30 min and then read at 595 nm (EnSpire, Perkin Elmer, Waltham, MA, USA). Titrated concentrations (0.5 to 20 nmol) of trolox were used for the calibration and the results were expressed as nmol equivalent of Trolox/mg of skin.

### 2.11. Evaluation of the Antioxidant Power by ABTS (2,2′-Azino-bis(3-ethylbenzothiazoline-6-sulfonic Acid)) Radical Sequestration Test

This method is based on the decay of ABTS^+^ radical cation color when it receives an electron from an antioxidant. For the ABTS reaction, 400 μL of 1.15% KCl were added to the samples followed by homogenization with the aid of Tissue-Tearor Biospec 985370 (Bartlesville, OK, USA) and centrifuged at 1000× *g* for 10 min at 4 °C, after which the supernatant was used for analysis. Measures of 7 μL of the supernatant and 200 μL of the diluted ABTS solution (7 mM of the ABTS solution with 2.45 mM potassium persulfate) were added to a plate. After 6 min, the absorbance was read at 730 nm (EnSpire, Perkin Elmer, Waltham, MA, USA) and compared with a standard curve of Trolox (0.01 to 20 nmol) and the results were expressed nmol of Trolox/mg of skin [41,50].

### 2.12. Evaluation of the Level of Endogenous Antioxidant: Reduced Glutathione (GSH)

GSH is considered the main marker for UVB-irradiation-induced oxidative stress because it is produced in high quantity by the epidermal cells [40]. Skin samples were diluted (1:4) in 0.02 M EDTA and triturated using Tissue Tearor-Biospec 985370 (Bartlesville, OK, USA). To the homogenate it was added 50% trichloroacetic acid in the ratio of 1: 0.2 EDTA and TCA, respectively. Then, the mixture was centrifuged twice at 2700× *g* at 4 °C for 10 min for the first time and 15 min and the final supernatant was removed for analysis. For quantitation of GSH levels, 50 μL of the supernatant was added to 100 μL of 0.4 M Tris buffer (pH 8.9) and 5 μL of a 1.9 mg/mL solution of DTNB in methanol was combined to the reaction. After 5 min of incubation, the samples were read at 405 nm (EnSpire, Perkin Elmer, Waltham, MA, USA), and the results were compared with a standard curve of titrated concentrations of GSH (5 to 150 μM) and expressed as μM GSH/mg skin [51].

### 2.13. Assay for Catalase Activity (CAT)

The principle of the technique is based on the decomposition of H_2_O_2_ followed by the decrease in absorbance. The difference in absorbance per unit of time measures the activity of the enzyme [52]. A volume of 500 μL of 0.02 M EDTA was added to skin samples prior to homogenization (Tissue Tearor-Biospec 985370; Bartlesville, OK, USA). Then, the homogenate was centrifuged twice at 2700× *g* for 10 min at 4 °C and the final supernatant was removed for analysis. For the reaction, 10 μL sample, 160 μL 1 M Tris-HCl buffer with 5 mM EDTA pH 8.0, 20 μL deionized water, and 20 μL of 200 mM H_2_O_2_ were added. The reaction was read 1 and 30 s after the addition of H_2_O_2_ at 240 nm (EnSpire, Perkin Elmer, Waltham, MA, USA). Data were expressed as catalase unit/mg skin/minute [52].

### 2.14. Evaluation of the Superoxide Anion Production

Superoxide anion is a free radical formed in the first reduction of oxygen to water during aerobic respiration and its production can be accentuated by UVB irradiation [53,54]. This method is based on the reduction of nitroblue tetrazolium (NBT) to formazan. Skin samples were homogenized with Tissue Tearor-Biospec 985370 (Bartlesville, OK, USA) in 0.02 M EDTA and centrifuged (2000× *g* for 20 s at 4 °C). First, 50 uL of the supernatant from each sample was added and incubated for 1 h. Subsequently, the supernatant was removed and NBT (1 mg/mL) was added to the medium. After 15 min of incubation, the supernatant was removed and 20 μL of 100% methanol was added to fix. Then, 120 μL of 2 M KOH and 140 μL of dimethylsulfoxide (DMSO) were added to solubilize the formazan compound (reduced NBT). The concentration of formazan formed was measured spectrophotometrically (EnSpire, Perkin Elmer, Waltham, MA, USA) at 620 nm and the results were presented as optical density (OD)/10 mg of skin [40].

### 2.15. Lipid Hydroperoxide (LOOH) Assay

The production of hydroperoxides is measured by a chemiluminescence method and lipoperoxidation is initiated by the addition of tert-butyl hydroperoxide, as previously described in [55] and adapted in [41]. Skin samples were collected and homogenized (Tissue Tearor-Biospec 985370, Bartlesville, OK, USA) in 800 μL of phosphate buffer (pH 7.4) and then centrifuged at 700× *g* for 2 min at 4 °C. For the assay, 250 μL of the supernatant was added to 1730 μL of reaction medium (120 mM KCl, 30 mM phosphate buffer pH 7.4) and 20 μL of 3 mM tert-butyl hydroperoxide. This assay was performed on a Beckman^®^ LS 6000 β counter (Fullerton, CA, USA) in a count range not coincident with the response between 300 and 620 nm. The reaction must be carried on in the dark at 30 °C for 120 min and the results were measured in counts per minute (cpm) per mg of skin.

### 2.16. Fluorescent Western Blot

Mice were terminally anesthetized 4 h after UVB radiation exposure, and the dorsal skin of hairless mice was collected in RIPA buffer containing protease and phosphatase inhibitors. The samples were ground, and the lysates were centrifuged (16,000× *g* for 20 min at 4 °C). The supernatant portion was transferred into a fresh tube and placed on ice for total protein quantitation (Bradford Reagent with BSA standard) and equalization. The protein extracts were separated by 10% SDS-PAGE and transferred onto a 0.2 μm nitrocellulose membrane (GE Healthcare-Amersham, Pittsburgh, PA, USA). Spectra Multicolor Broad Range and PageRuler Prestained Protein Ladders (Thermo Scientific, Waltham, MA, USA) were used for molecular weight confirmation. Membranes were blocked for one hour in 5% BSA TBS (blocking solution) with gentle shaking, followed by overnight incubation in primary antibodies (Table 1). The membranes were then washed in TBS three times for ten minutes, incubated with secondary fluorescent antibodies (Table 1) for an hour, and the washing step with TBS was repeated. Image acquisition was performed using the Amersham ImageQuant 800 fluor (Cytiva, Marlborough, MA, USA). The optical density of the bands was quantitated using the Image LabTM Software, version 6.1 (Bio-Rad Laboratories, Hercules, CA, USA).

### 2.17. mRNA Expression of Cybb (gp91^phox^) and Cox2 by RT-qPCR

The mRNA quantitative analyses for the *Cybb* (gp91^phox^) and *Cox2* (COX-2) genes were carried out through the reverse transcription quantitative polymerase chain reaction (RT-qPCR). RNA from the dorsal skin of hairless mice was extracted using lysis reagent QIAZOL (Qiagen, Aarhus, Denmark) following the manufacturer’s instructions. RNA concentration and purity were confirmed spectrophotometrically, (260/280 ratio: ≈2.0 and 260/230 ratio: 1.6 to 2.2). A QuantiTect Reverse Transcription Kit (Qiagen, Aarhus, Denmark) was used to transcribe the mRNA into complementary DNA (cDNA) and QuantiNova SYBR Green PCR Kit (Qiagen, Aarhus, Denmark) for quantitative real-time PCR analysis. The employed primer sequences are available in Table 1 (attached to Fluorescent Western Blot Methods—item 2.17.). The comparative 2−(ΔΔCt) method was used to determine the relative gene expression to the β-actin reference gene (*Actb*) [56].

### 2.18. Statistical Analysis

The GraphPad Prism 7 software (GraphPad Software Inc., San Diego, CA, USA) was used for the statistical analyses. Data were considered significantly different at *p* < 0.05 upon assessment applying one-way analysis of variance (ANOVA) considering that, in all experiments, there were at least 3 groups for comparison. The Tukey multiple comparisons test was used for post hoc analyses. Results were presented as mean ± standard error (SEM). Measurements made with 6 animals in each group per experiment and are representative of 2 separate experiments.

## 3. Results

### 3.1. RvD2 Reduces UVB-Irradiation-Induced Skin Edema and Myeloperoxidase (MPO) Activity

**Figure 1:**
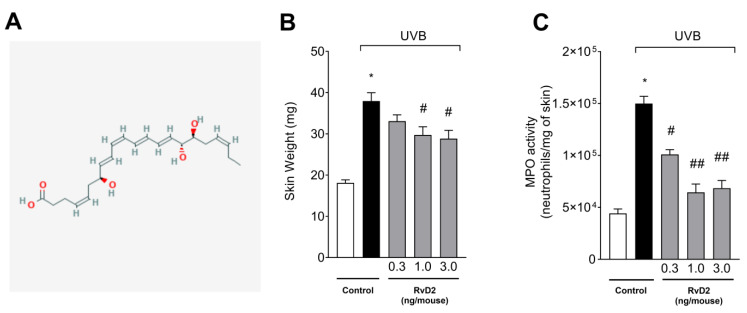
RvD2 reduces skin edema and MPO activity triggered by UVB irradiation. The time point of sample collection was 12 h after the end of irradiation. (**A**) RvD2 structure (2D) available in PubChem [NLM PubChem [Internet]. Bethesda (MD): National Library of Medicine (US), National Center for Biotechnology Information; 2004-. PubChem Compound Summary for CID 11383310, Resolvin D2; [accessed on 27 June 2025]. Available from: https://pubchem.ncbi.nlm.nih.gov/compound/Resolvin-D2]. (**B**) Skin edema and (**C**) and MPO activity. Bars represent means ± SEM of 6 mice per group and represent two separate experiments. Statistical analysis was performed by one-way ANOVA followed by Tukey’s test. [* *p* < 0.05 compared to the non-irradiated group; # *p* < 0.05 compared to the irradiated group (vehicle); ## *p* < 0.05 compared to the irradiated group and group treated with the dose of 0.3 ng/mouse].

Figure 1A presents the RvD2 structure [57]. Exposure to UVB irradiation resulted in skin edema and increased MPO activity in the irradiated group compared to the non-irradiated group. These inflammatory responses, e.g., skin edema and increase in MPO activity, are characteristic in UVB skin inflammation [14,58]. The treatment with RvD2 reduced edema and MPO activity when compared with the irradiated group. There was a reduction in cutaneous edema with the doses of 1.0 and 3.0 ng/mouse and MPO activity with the three doses tested (0.3, 1.0 and 3.0 ng/mouse) when compared to the irradiated group (Figure 1B,C).

### 3.2. RvD2 Inhibits UVB Irradiation-Triggered MMP-9 Enzymatic Activity

**Figure 2:**
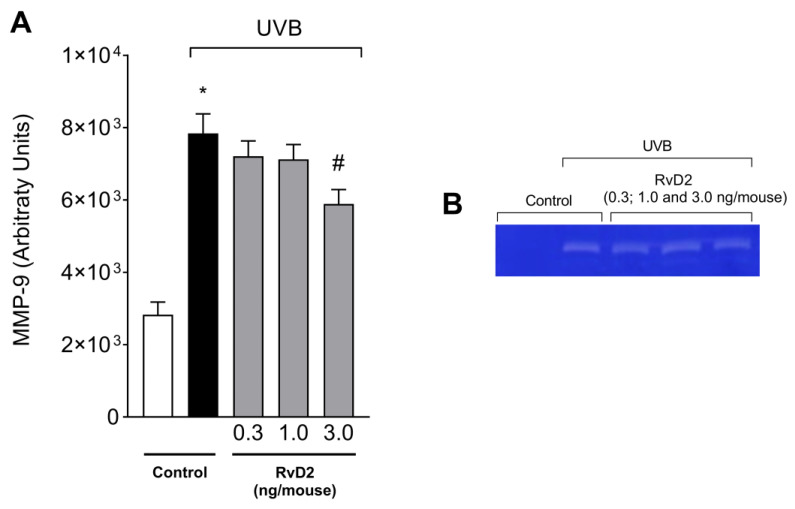
RvD2 reduces UVB-irradiation-induced MMP-9 activity. The time point of sample collection was 12 h after the end of irradiation. MMP-9 activity (**A**) and representative gelatin zymography image (**B**). Bars represent means ± SEM of 6 pools of samples per group and each pool contained the samples of 2 mice. One-way ANOVA followed by Tukey’s post hoc test. [* *p* < 0.05 compared to the non-irradiated group; # *p* < 0.05 compared to the irradiated group (vehicle)].

UVB irradiation increased the activity of MMP-9 compared to the non-irradiated group. MMP-9 is responsible for the degradation of extracellular matrix that occurs upon the UVB irradiation of the skin [39,59]. The treatment with RvD2 significantly reduced enzyme activity with the dose of 3.0 ng/mouse as can be observed in the semi-quantitative data (Figure 2A) and representative image (Figure 2B). The results of Figure 1 and Figure 2, in which only the dose of 3.0 ng/mouse was sufficient for inhibiting all these three inflammatory parameters (e.g., skin edema, MPO activity, and MMP-9 activity), were the basis for the decision to select this dose of RvD2 for the next experiments; in these, we approached the pathological changes in the skin and the mechanisms of action of RvD2. This is a rational approach for limiting the number of animals used in the study.

### 3.3. RvD2 Decreases LysM-eGFP^+^ Cells Recruitment Triggered by UVB Irradiation

**Figure 3:**
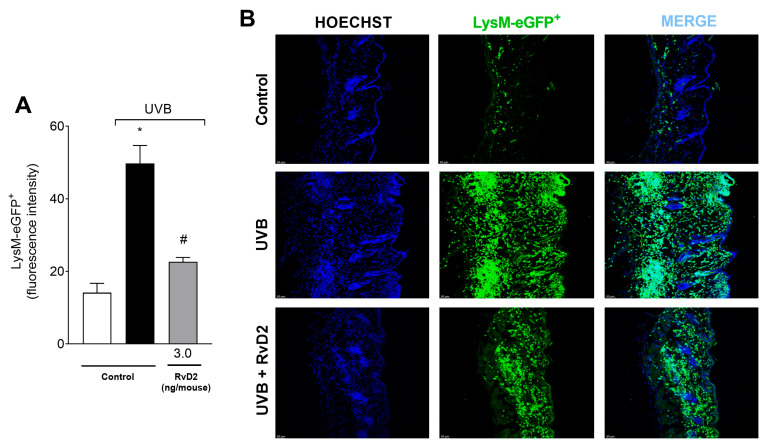
RvD2 minimizes UVB-irradiation-induced neutrophil infiltration. LysM-eGFP^+^ cell recruitment was evaluated in samples collected 12 h after UVB exposure. Results are expressed in eGFP fluorescence intensity (**A**). Representative images of the three groups (**B**) described in panel (**A**). Bars (**A**) are means ± SEM of 6 mice per group per experiment and represent two separate experiments. One-way ANOVA followed by Tukey’s post hoc test. [* *p* < 0.05 compared to the non-irradiated group and # *p* < 0.05 compared to the irradiated group (vehicle)].

To evaluate the anti-inflammatory activity of RvD2 against photo-oxidative injury, we investigated neutrophil/macrophage migration using a LysM-eGFP^+^ reporter mouse. Lysozyme is expressed by neutrophils and macrophages [60]. The findings revealed that systemic administration of RvD2 (3.0 ng/mouse) attenuated this infiltration of LysM^+^ cells, reducing the inflammatory effects of UVB as observed in the semi-quantitative analyses (Figure 3A) and representative images (Figure 3B).

### 3.4. RvD2 Reduces UVB-Irradiation-Induced Mast Cell Counts

**Figure 4:**
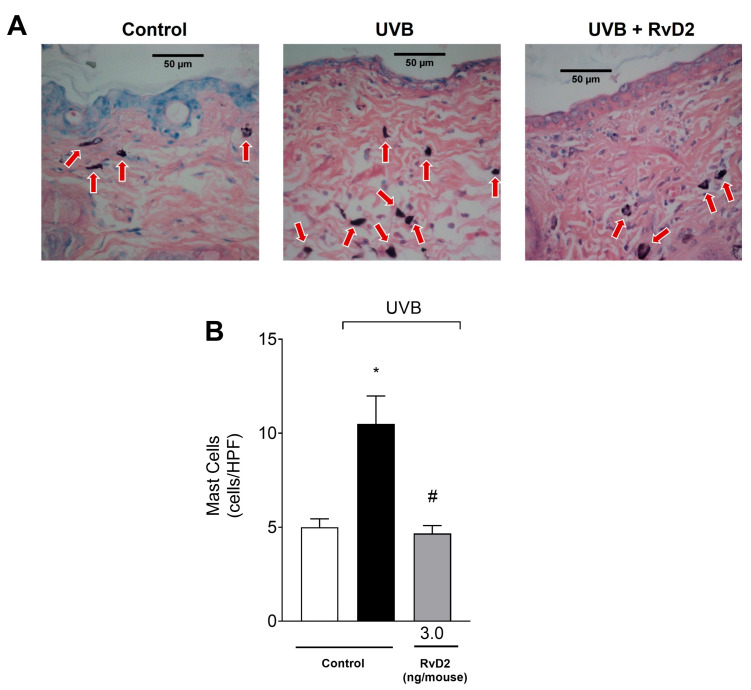
RvD2 reduces UVB-irradiation-induced mast cell counts. Sections stained with blue toluidine were examined using light microscopy at 40× magnification (**A**) and the number of mast cells (indicated by red arrows) were (**B**) counted. The time point of sample collection was 12 h. Bars represent means ± SEM of 6 mice per group per experiment and represent two separate experiments. One-way ANOVA followed by Tukey’s post hoc test. [* *p* < 0.05 compared to the non-irradiated group and # *p* < 0.05 compared to the irradiated group (vehicle)].

After exposure to UVB irradiation, there was a significant increase in the number of dermal mast cells, which are cells participating in the immune response [61]. Furthermore, samples from animals treated with RvD2 (3.0 ng/mouse) showed a reduction in the counts of these cells when compared to samples from the irradiated control group, as observed in the representative images (Figure 4A) and total counts (Figure 4B).

### 3.5. RvD2 Reduces UVB-Irradiation-Induced Inflammatory Parameters by Decreasing the Epidermal Thickness and Sunburn Cell Formation

**Figure 5:**
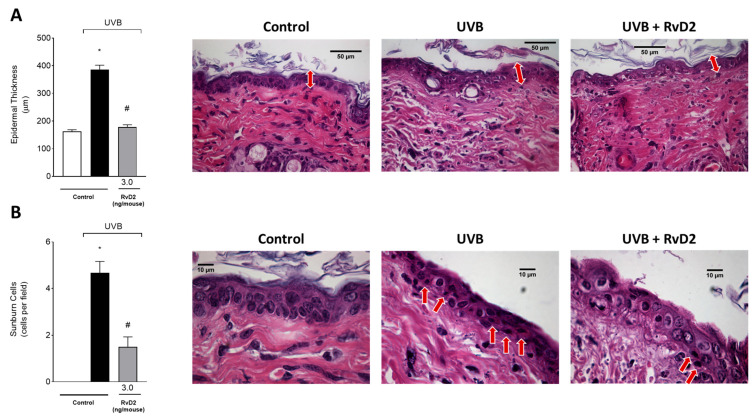
RvD2 reduces UVB-irradiation-induced inflammatory parameters by decreasing the epidermal thickness and sunburn cells count. Epidermal thickness (**A**) and sunburn cells (**B**). The time point of sample collection was 12 h. Staining was hematoxylin and eosin. Epidermal thickness (μm; indicated by double point red arrows) ((**A**); 40×)) and the number of sunburn cells ((**B**); indicated by red arrows; 100×)). Bars represent means ± SEM of 6 mice per group per experiment and represent two separate experiments. One-way ANOVA followed by Tukey’s post hoc test. [* *p* < 0.05 compared to the non-irradiated group and # *p* < 0.05 compared to the irradiated group (vehicle)].

The epidermal thickness of the dorsal skin had a significant increase in the group exposed to UVB irradiation compared to the non-irradiated control group. In contrast, treatment with RvD2 (3.0 ng/mouse) was able to reduce this epidermal hypertrophy (Figure 5A). Furthermore, another consequence of exposure to UVB irradiation is the presence of sunburn cells. These cells present with eosinophilic cytoplasm and condensed nucleus because they are apoptotic keratinocytes [62]. Sunburn cells were observed in greater numbers in the irradiated group than in the non-irradiated group, and RvD2 reduced their counts (Figure 5B).

### 3.6. RvD2 Reduces UVB-Irradiation-Induced Damage to Collagen Fibers

**Figure 6:**
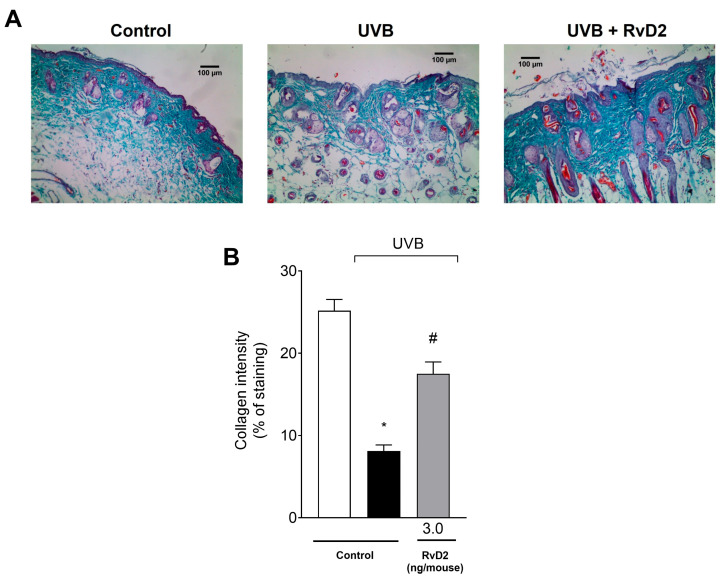
RvD2 reduces UVB-irradiation-induced collagen fiber damage. The time point of sample collection was 12 h. Collagen fiber formation was evaluated using Masson’s trichrome staining (**A**). The Image J Program (100× magnification) was applied to analyze collagen fiber intensity and bundles shown in blue (**B**). Bars represent means ± SEM of 6 mice per group per experiment and represent two separate experiments. One-way ANOVA followed by Tukey’s post hoc test. [* *p* < 0.05 compared to the non-irradiated group and # *p* < 0.05 compared to the irradiated group (vehicle)].

To enable the visualization of the collagen fibers [63], the epidermal tissue sections were stained with Masson’s trichrome. In this staining, a decrease in their color intensity was observed in the irradiated control group compared to the non-irradiated control group. Treatment with RvD2 (3.0 ng/mouse) prevented UVB damage to collagen fibers, as observed in the representative images (Figure 6A) and the collagen intensity assessment (Figure 6B).

### 3.7. RvD2 Inhibits Skin Inflammation Reducing Cytokine Production Caused by UVB Irradiation

**Figure 7:**
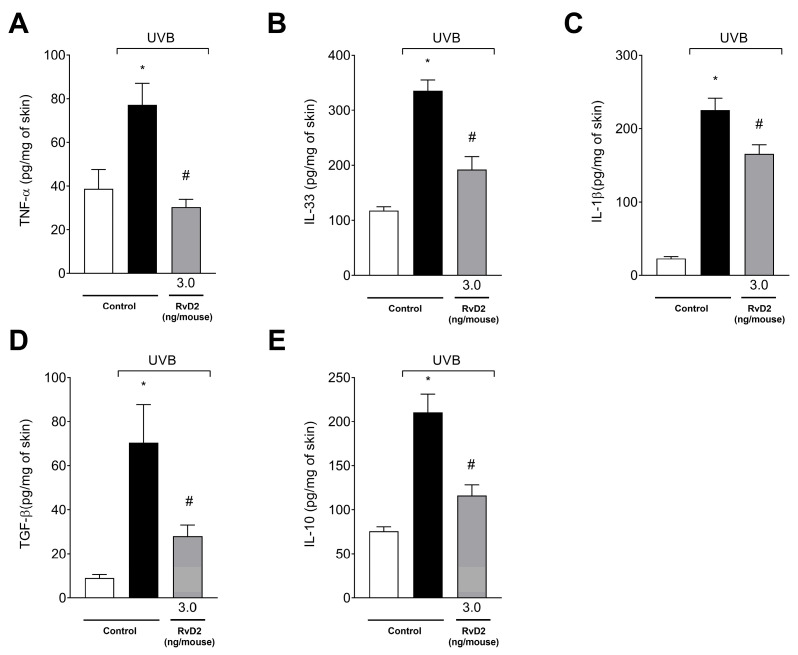
RvD2 inhibits UVB-irradiation-induced proinflammatory cytokines TNF-α (**A**), IL-33 (**B**), and IL-1β (**C**) and anti-inflammatory cytokines TGF-β (**D**) and IL-10 (**E**), and production induced by UVB irradiation. The time point of sample collection was 4 h. Bars represent means ± SEM of 6 mice per group and represent two separate experiments. One-way ANOVA followed by Tukey’s post hoc test. [* *p* < 0.05 compared to the non-irradiated group; # *p* < 0.05 compared to the irradiated group (vehicle)].

The results demonstrated that there was a significant increase in the production of all cytokines evaluated upon exposure to UVB irradiation, including the proinflammatory TNF-α (Figure 7A), IL-33 (Figure 7B) and IL-1β (Figure 7C) and the anti-inflammatory agents TGF-β (Figure 7D) and IL-10 (Figure 7E). Treatment with RvD2 (3.0 ng/mouse) was able to inhibit the increase in these cytokines (Figure 7A–E).

### 3.8. RvD2 Optimizes the Skin’s Antioxidant Profile Modification Caused by UVB Irradiation

**Figure 8:**
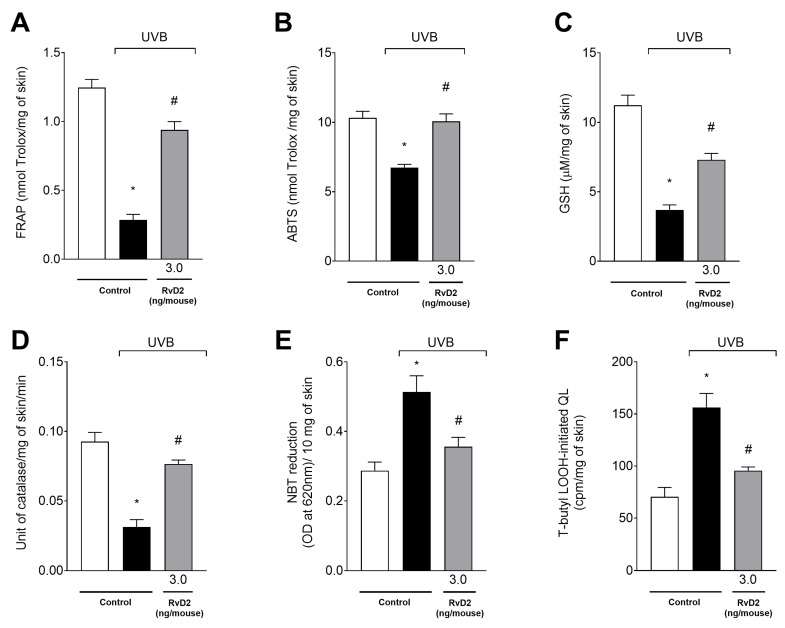
RvD2 maintains antioxidant capacity and reduces the production of superoxide anion and hydroperoxides (LOOH). FRAP ((**A**); 12 h), ABTS ((**B**); 12 h), GSH ((**C**); 12 h), catalase activity ((**D**); 2 h), superoxide anion production ((**E**); 2 h), and lipid hydroperoxides production ((**F**); 4 h) were assessed at indicated time points. Bars represent means ± SEM of 6 mice per group per experiment and represent two separate experiments. One-way ANOVA followed by Tukey’s post hoc test. [* *p* < 0.05 compared to the non-irradiated group; # *p* < 0.05 compared to the irradiated group (vehicle)].

The antioxidant capacity was evaluated initially through FRAP (Figure 8A) and ABTS (Figure 8B) assays and the dosage of the endogenous antioxidant GSH (Figure 8C). It was observed that UVB-irradiation-induced exposure was able to significantly reduce all the parameters tested compared with the non-irradiated group. In addition, treatment with RvD2 was able to inhibit the depletion of skin antioxidant capacity with significant effects with a dose of 3.0 ng/mouse. On the other hand, the doses of 0.3 and 1.0 ng/mouse did not maintain the antioxidant capacity of the skin as shown through the results of all the parameters analyzed (Figure 8A–C); this comes with the exception of the dose of 1.0 ng/mouse in the FRAP assay that also induced significant antioxidant activity. Therefore, 3.0 ng/mouse was selected as the dose of choice for the next set of tests.

UVB irradiation reduced CAT activity (Figure 8D) and increased both superoxide anion (NBT assay) (Figure 8E) and LOOH (Figure 8F) production in the irradiated group relative to the non-irradiated group. Treatment with RvD2 (3.0 ng/mouse) significantly inhibited the depletion of the CAT assay and the production of superoxide anion and hydroperoxides when compared to the irradiated group (Figure 8D–F).

### 3.9. RvD2 Inhibits NF-κB Pathway Activation and Downstream It Targets mRNA Expression Caused by UVB Irradiation

**Figure 9:**
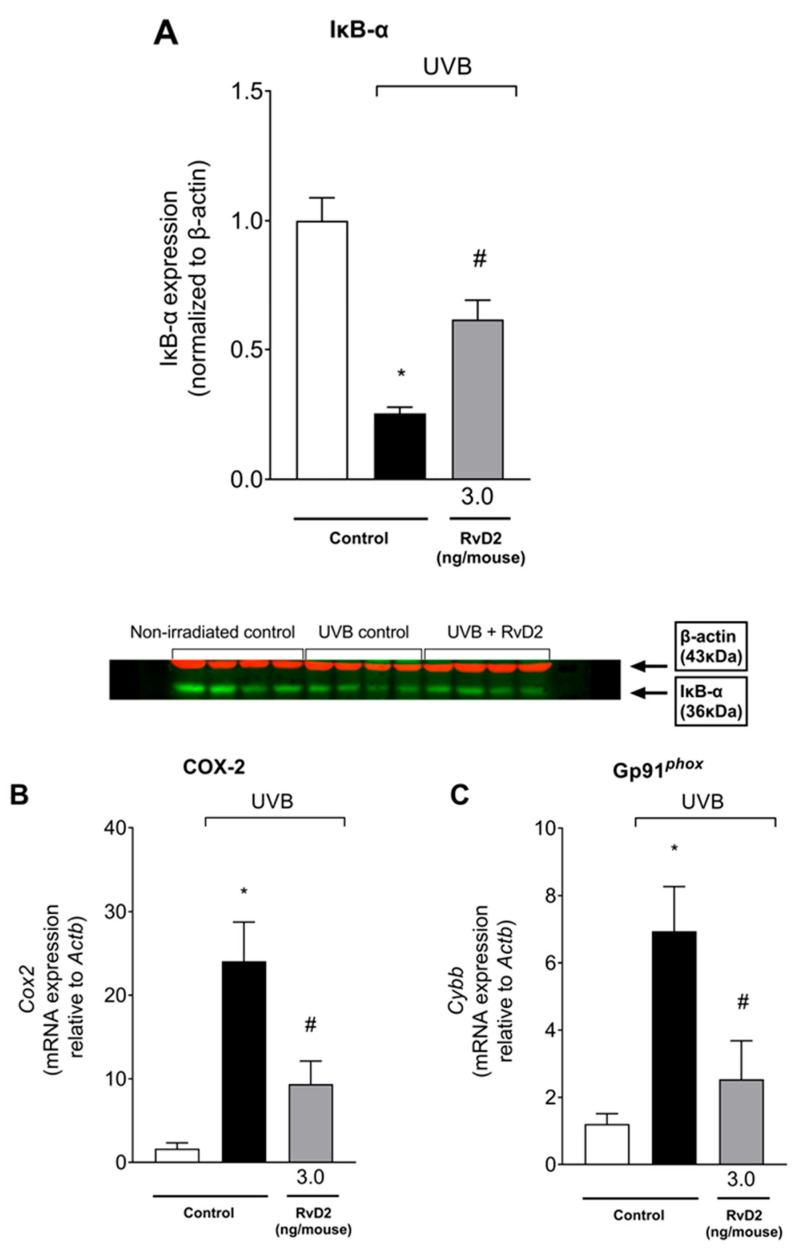
RvD2 inhibits the activation of NF-κB pathway, and of its downstream targets triggered by UVB irradiation. Degradation of IκB-α was observed 4 h after irradiation as presented by the band intensity data and Western blot image (**A**). COX-2 (**B**) and gp91^phox^ (**C**) mRNA expression 4 h after UVB irradiation. Bars represent means ± SEM of 4 (Western Blot) or 6 (RT-qPCR) mice per group per experiment and represent two separate experiments. One-way ANOVA followed by Tukey’s post hoc test. [* *p* < 0.05 compared to the non-irradiated group; # *p* < 0.05 compared to the irradiated group (vehicle)].

In order to further comprehend the mechanisms underlying RvD2 protective effects against UVB-induced skin pathology, the protein levels of IκB-α that acts by binding to the p65/p50 NF-κB dimers keeping the complex inactive [64] were assessed using Western Blot assay. The activation of the NF-κB pathway is typical on UVB-induced skin damage [65] and can be observed through an increased degradation of IκB-α. Once IκB-α is degraded, active NF-κB undergoes nuclear translocation and subsequent DNA binding, acting as a transcription factor inducing a proinflammatory expression profile [66]. Four hours after UVB radiation stimulus, the protein levels of IκB-α were reduced, indicating its degradation, while treatment with RvD2 was able to impair such degradation, therefore indicating an inhibition in NF-κB pathway activation (Figure 9A). COX-2 is an enzyme that produces proinflammatory prostanoids and superoxide anions as a byproduct [67]. The gp91^phox^ is a catalytic subunit of NADPH oxidase, an enzyme that generates superoxide anion from oxygen (O_2_^−^), acting as an important source for ROS production [68]. COX-2 and gp91^phox^ mRNA expression depend on NF-κB activation. In line with the mechanism of RvD2 of inhibiting NF-κB activation, RvD2 also downregulated COX-2 (Figure 9B) and gp91^phox^ (Figure 9C) mRNA expression. Thus, RvD2 reduces the activation of the NFκB pathway and the mRNA expression of its downstream targets, which further supports this mechanism.

## 4. Discussion

UV irradiation is an external factor that can generate skin pathological modifications, including DNA damage that can lead to skin cancer [54,69]. It is agreed that the pathophysiological mechanisms triggered by UVB irradiation involve the association between oxidative stress and the production of inflammatory mediators [70]. It should also be noted that the use of sunscreens is not fully effective against UVB radiation. Sunscreens can even further promote the generation of ROSs and cause allergic reactions [71,72]. The antioxidant system that protects the skin against oxidative damage is deficient under stress conditions; therefore, protecting the skin with antioxidants or anti-inflammatories would be a valuable addition [13,14]. Pro-resolution lipid mediators are generated to attenuate the amplification of the inflammatory response and consequently that of ROSs, stimulating healing and tissue regeneration [73,74,75]. RvD2 is a lipid mediator derived from the enzymatic conversion of DHA, which has shown therapeutic potential in several other disease models [14,15,16,17,18,19,20,21,22,23,24,25,26,27].

In the present study, the systemic treatment with RvD2 was found to protect the skin against UVB-irradiation-induced inflammatory damage, significantly reducing the following parameters analyzed: edema, MPO, MMP-9 activity, LysM^+^ cells infiltration, collagen fiber degradation, epidermal thickness, number of mast cells, and sunburn cell formation. RvD2 anti-inflammatory action is also accompanied by a reduction in the production of proinflammatory cytokines, such as TNF-α, IL-33, and IL-1β, responsible for upregulating the immune response [76,77,78]. The cytokines TGF-β and IL-10 have a role in limiting inflammation. For instance, evidence supports the co-release of IL-10 with proinflammatory cytokines to limit the inflammatory reaction as an anti-inflammatory mechanism [79,80]. The concomitant inhibition of pro- and anti-inflammatory cytokines suggests that RvD2 blocked the inflammatory process in a step before their production. This is likely due to an inhibition of inflammation development, which made the release of anti-inflammatory cytokines that would counteract inflammation unnecessary.

Other studies with different experimental models have shown that RvD2 reduces inflammation, corroborating our findings. In cecal ligation and puncture, a model of microbial peritonitis sepsis that evolves to sepsis, treatment with RvD2 was able to inhibit leukocyte recruitment, which was explained by its effect on cytokine production. RvD2 decreases proinflammatory cytokines production, including TNF-α and IL-1β, and increases the production of anti-inflammatory IL-10, resulting in diminished tissue damage [38]. MPO activity was also evaluated in a model of experimental colitis in mice as an indirect test for the analysis of neutrophil infiltration, and its activity was found to be reduced by RvD2 treatment [81]. Excessive exposure to UVB irradiation promotes the synthesis of cytokines that recruit neutrophils producing enzymes such as MMP and MPO; these enzymes are involved in the degradation of collagen fibers and the additional production of ROSs, respectively [82,83,84]. Another inflammatory response caused by UVB irradiation is the formation of edemas to which cytokine, COX-2-derived prostanoids, and ROSs can be accounted [85,86,87]; therefore, the RvD2 inhibition of those targets might explain its anti-edematogenic activity.

The production of cytokines triggers other cellular events. Keratinocytes are epidermal cells that have adaptive mechanisms for enhancing protection against UVB irradiation. One of these mechanisms is the proliferation of the cells at the site of the lesion, resulting in increased epidermal thickness and skin edema [2,42,88]. Upon the evolution of the damage caused by UV irradiation, keratinocytes undergo apoptosis, creating sunburn cells that are characterized microscopically as cells with pyknotic nuclei and eosinophilic cytoplasms [89,90]. These data are consistent with our findings reflecting the reduction of these cells with treatment with RvD2 at a dose of 3.0 ng/mouse. Corroborating these findings, RvD1, another DHA-derived metabolite, was responsible for decreasing these inflammatory parameters at a dose of 30 ng/mouse in a UVB-induced skin lesion model [14]. Thus, RvD2 presents similar outcomes in UVB skin inflammation, but with a 10-fold lower dose.

UVB irradiation generates ROSs, such as superoxide anion, which can react with lipids, promoting an event called lipid peroxidation [70,91]. Furthermore, increased production of ROSs leads to an imbalance in the antioxidant system, resulting in the depletion of molecules belonging to this system, such as GSH and CAT [12,92]. GSH neutralizes oxidants by the action of the sulfhydryl group of molecules, and CAT converts hydrogen peroxide to water (H_2_O) and oxygen (O_2_), preventing the formation of toxic radicals such as hydroxyl [93]. In this work, the systemic i.p. treatment with RvD2 was able to significantly reduce oxidative stress triggered by UVB irradiation by lessening superoxide anion and hydroperoxide production, downregulating gp91^phox^ expression, as well as maintaining the levels of the GSH and CAT endogenous antioxidants. In addition, RvD2 protected the skin against oxidative damage through its ferric reduction ability and the ABTS radical parameters were evaluated for the overall antioxidant capacity of the tissue [50].

Resolvins, in general, have been demonstrated to induce antioxidant activity in other experimental models; for instance, in a model of osteoarthritis, an improvement of the antioxidant capacity was observed through maintaining the basal levels of GSH in the group treated with RvD1 [94]. In another work, RvD2 was able to reduce oxidative stress by decreasing the levels of carbonylated proteins in a cigarette smoke exposure model [95,96]. Treatment with RvD2 decreased TNFα-induced superoxide anion production in mouse aortic smooth muscle cell. Inflammatory cytokines such as TNF-α are mediators that intensify the local response, resulting in the additional production of ROSs and the amplification of tissue damage [96].

NF-κB is known to be one of the transcription factors that regulates the genes that are expressed upon UVB stimulus. Once cells suffer DNA and molecular damage caused by UVB, NF-κB initiates the upregulation of inflammatory gene expression [64]. Here, we observed that RvD2 inhibited IκB-α degradation, which is a surrogate marker of the activation of the NF-κB pathway. This mechanism is aligned with an inhibition of oxidative stress and cytokine production. As an inflammatory beacon downstream of NF-κB, the expression of cyclooxygenase-2 (COX-2), an enzyme with peroxidase and cyclooxygenase active sites essential to prostaglandin biosynthesis, showed increased mRNA expression as a result of UVB. Previous studies have shown that COX-2 expression plays a key role in mouse skin upon irradiation with UVB [97], and its increased expression can be observed after a single exposure [98]. Our findings further confirm the induction of COX-2 mRNA expression due to UVB exposure, a pathologic event that was inhibited after RvD2 treatment. Interestingly, topical treatment with docosahexaenoic acid (DHA), a precursor of D-series resolvins, maresins, and protectins, has also been shown to inhibit NF-κB together with reduced COX-2 mRNA expression, protecting the skin of hairless mice against UVB-induced oxidative stress and inflammation [99]. These data might indicate that enzymes involved in SPM synthesis are present in UVB-irradiated skin and the skin is likely to receive SPM treatment in this pathological condition. The downregulation of NF-κB in vitro has been shown to enhance UV-induced apoptosis [100], suggesting the possibility that RvD2 reduction in sunburn cell formation could also be explained by NF-κB inhibition.

Regarding the effects of RvD2 that are dependent on the targeting of NF-κB induction of oxidative stress, it is worth noticing that NF-κB itself has been highlighted as a key factor in controlling NADPH oxidase expression and function; this has been shown in the increased expression of the NADPH oxidase subunit gp91^phox^ [8]. The activity of RvD2 in reducing gp91^phox^ expression is particularly interesting due to the role of the NADPH oxidase enzyme, which is specifically devoted to the production of superoxide anion, a key player in cell signaling and tissue injury [66].

Recently, we demonstrated that RvD1 reduces UVB-irradiation-induced skin oxidative stress, because it improved the antioxidant system (GSH and CAT assays) and inhibited hydroperoxide formation and superoxide anion production [14]. However, it should be noted that the antioxidant and anti-inflammatory activities of RvD1 were demonstrated at a dose of 30 ng/mouse. RvD5 also presents similar activity at the dose of 30 pg/mouse [45] the aspirin-triggered RvD1 is active at the dose of 300 pg/mouse [101] and the aspirin-triggered LXA4 is active at the dose of 3 ng/mouse [102]. Thus, RvD2 is active in an intermediate dose (3 ng/mouse) compared to other SPMs in the UVB irradiation skin exposure model. Prior evidence has demonstrated the following findings: RvD1 and aspirin-triggered RvD1 are agonists of GPR32 and ALX/FPR2 receptors; aspirin-triggered LXA4 and LXA4 are also agonists of ALX/FPR2 receptors; RvD5 binds and activates GPR101; RvD2 is an agonist of GPR18 [103]. The activation of the GRP18 receptor promotes the phagocytosis of apoptotic cells and the removal of cell debris for the resolution of inflammation [104].

On the other hand, recent evidence demonstrates that DHA-derived SPMs, such as RvD1, RvD2, and RvD5, can also function as biased allosteric modulators during the prostaglandin E2 (PGE2) activation of EP4 signaling with anti-inflammatory consequences; in addition, they can shift the EP4 receptor towards the promotion of phagocytosis. RvD1 and RvD5, but not RvD2, can activate human EP4 receptors. RvD1 and RvD5, but not RvD2, were found to be capable of displacing PGE2 binding to EP4 [105]. These differences in the mechanisms of action of RvD1, RvD2, and RvD5 suggest that the mechanisms of RvD2 could be considered to be unique among the three resolvins. Adding to the complexity in this area, the same study challenged the notion that SPMs are agonists of the GPR32, GPR18, and ALX/FPR2 receptors; the authors presented data that demonstrate that they act solely through the allosteric modulation of EP4 receptors and/or affecting PGE2 binding to EP4 [105]. This novel information means that it is difficult to speculate as to which SPM would be more promising than others; further comprehension of their receptor-related mechanisms seem necessary to reach any such conclusions.

The results of the present study were obtained through applying an acute inflammation model; endogenous resolution mechanisms are likely not fully active at the included time points because BOC-2, an ALX/FPR2 antagonist, did not affect the course of inflammation triggered by UVB irradiation [102]. It is notable that, while some SPMs would be agonists of ALX/FPR2, there are also other agonists of this receptor, such as the peptide annexin-1, that are involved in inflammation resolution [106]. RvD2 presented anti-inflammatory actions such as inhibiting NF-κB activation, as observed in the increased levels of IκBα. This activity resulted in diminished mRNA expression of enzyme or enzyme catalytic subunits involved in inflammation and oxidative stress, aligning with the observation of a reduction in inflammation and oxidative stress, and of tissue pathology. NF-κB activation represents upstream COX-2 and gp91^phox^ expression and cytokine production. The evidence supports the idea that, in the case of lipopolysaccharide stimulation of macrophages, as well as in the in vivo stimulation of zymosan or *Escherichia coli*, a downregulation loop occurs in which COX-2-derived PGE2 activates EP4 to reduce NF-κB activation [93,95]. This loop could limit the induction of COX-2, cytokines, and other molecules whose expressions are regulated by NF-κB. Therefore, these results [93,95] are in line with the proposed RvD2 allosteric modulation of PGE2 signaling via EP4 receptors [93], and would explain the inhibition of inflammatory mechanisms and skin pathologies by treatments with RvD2.

## 5. Conclusions

The results of the present study demonstrate, for the first time, that RvD2 reduces the disease parameters that are related to inflammation and oxidative stress caused by UVB irradiation in the skin. We proceed by demonstrating the biological activities of RvD2 that are dependent on targeting oxidative stress, enhancing antioxidant mechanisms, reducing inflammation, and, more specifically, reducing NF-κB activation, as observed in the reduction in IκB-α degradation. Therefore, although RvD2 is classified within the pro-resolution class of lipid mediators [107], under the experimental conditions of the present study, its mechanisms of action depended mostly on the stimulation of antioxidant responses and the inhibition of inflammatory mechanisms.

## Figures and Tables

**Table 1:** Specifications regarding antibodies and primer sequences employed.

Antibodies Used for Fluorescent Western Blot						
Target	Primary Antibody	Dilution	Supplier/Cat No.	Secondary Antibody	Dilution	Supplier/Cat No.
IκB-*α*	IκBα (L35A5) Mouse mAb (Amino-terminal Antigen)	1:1000	Cell Signaling/4814	Goat anti-Mouse IgG (H+L) Cross-Adsorbed Secondary Antibody, Alexa Fluor™ 488	1:1000	Invitrogen/A11001
β-actin	β-Actin (D6A8) Rabbit mAb	1:1000	Cell Signaling/8457	Goat anti-Rabbit IgG (H+L) Cross-Adsorbed Secondary Antibody, Alexa Fluor™ 790	1:1000	Invitrogen/A113667
Primer sequences for RT-qPCR						
*Gene* (Target)	Forward sequence (5′ to 3′)			Reverse sequence (5′ to 3′)		
*Cybb* (Gp91^phox^)	AGC TAT GAG GTG GTG ATG TTA GTG G			CAC AAT ATT TGT ACC AGA CAG ACT TGA G		
*Cox2* (COX-2)	GTG GAA AAA CCT CGT CCA GA			GCT CGG CTT CCA GTA TTG AG		
*Actb* (β-actin)	AGC TGC GTT TTA CAC CCT TT			AAG CCA TGC CAA TGT TGT CT		

## Data Availability

The data that support the findings of this study are available from the corresponding author upon reasonable request.
